# Improving the diagnosis of radiation necrosis after stereotactic radiosurgery to intracranial metastases with conventional MRI features: a case series

**DOI:** 10.1186/s40644-022-00470-6

**Published:** 2022-07-06

**Authors:** Arian Lasocki, Joseph Sia, Stephen L. Stuckey

**Affiliations:** 1 Department of Cancer Imaging, Peter MacCallum Cancer Centre, Grattan St, Melbourne, VIC 3000 Australia; 2 Sir Peter MacCallum Department of Oncology, The University of Melbourne, Parkville, VIC Australia; 3 Department of Radiology, The University of Melbourne, Parkville, VIC Australia; 4 Department of Radiation Oncology, Peter MacCallum Cancer Centre, Melbourne, VIC Australia; 5 School of Clinical Sciences at Monash Health, Monash University, Clayton, VIC Australia; 6 Department of Medical Imaging and Radiation Sciences, Monash University, Clayton, VIC Australia

**Keywords:** Intracranial metastases, Magnetic Resonance Imaging, Radiation necrosis, Stereotactic radiosurgery

## Abstract

**Background:**

The distinction between true disease progression and radiation necrosis after stereotactic radiosurgery to intracranial metastases is a common, but challenging, clinical scenario. Improvements in systemic therapies are increasing the importance of this distinction. A variety of imaging techniques have been investigated, but the value of any individual technique is limited.

**Case presentation:**

Assessment should extend beyond simply the appearances of the lesion at a given timepoint, but also consider local anatomy and lesion evolution. Firstly, enlargement of a metastasis is affected by local anatomical boundaries, such as the dural reflections or cerebrospinal fluid spaces. In contrast, the radiation dose administered with stereotactic radiosurgery does not respect these anatomical boundaries and is largely concentric around the treated lesion. Therefore, new, non-contiguous enhancement across such a boundary can be confidently attributed to radiation necrosis. Secondly, the dynamic nature of radiation necrosis may result in a change in lesion shape, with different portions of the lesion simultaneously enlarging and regressing. Regression of part of a lesion indicates radiation necrosis, even if the overall lesion enlarges. This case series describes these two features and provides illustrative clinical examples in which these features allowed a confident diagnosis of radiation necrosis.

**Conclusions:**

The distinction between true disease progression and radiation necrosis should extend beyond just the appearances of the lesion. More nuanced interpretation incorporating a relationship to anatomical boundaries and a change in shape can improve accurate diagnosis of radiation necrosis.

## Background

Intracranial metastases (IM) are a frequent challenge in managing patients with metastatic disease. While IM can respond to systemic therapies [1], intracranial progression despite extracranial response remains a common clinical scenario. Radiotherapy (RT), in particular stereotactic radiosurgery (SRS), provides an effective means of treating IM, with high rates of local control [2]. The main limitation of SRS, however, is the risk of radiation necrosis (RN) affecting adjacent brain tissue, which occurs in 5–26% of lesions treated, most commonly 6–11 months after SRS [3]. In addition to managing symptoms associated with RN, it is important to distinguish RN from true tumor progression (progressive disease, PD), as the management of these two entities differs substantially. It is expected that RN will regress spontaneously over time, though medical treatments (for example, corticosteroids or bevacizumab) or even surgical resection may be warranted for control of symptoms. Therefore, additional treatment due to an incorrect diagnosis of PD (for example, surgical resection, repeat SRS or a change in medical therapy) can lead to significant unnecessary morbidity. Similarly, a delayed diagnosis of PD may lead to worse patient outcomes, for example a need for more extensive surgical resection.

Accurate diagnosis of RN is notoriously challenging, however, due to an overlap in the conventional MRI (Magnetic Resonance Imaging) appearances with PD [4]. This has led to the investigation of a variety of advanced MRI sequences, including Diffusion-Weighted Imaging, Perfusion and Spectroscopy [4]. Zach et al. reported promising results with delayed-contrast MRI [5], though subsequent validation and clinical implementation have been limited at this stage. PET (positron emission tomography) using amino acid tracers such as FET (fluorine-18-fluoroethyl-L-tyrosine) may also be used [6, 7]. Nevertheless, each technique has its limitations, prompting more research into the augmentation of traditional imaging interpretation with artificial intelligence (AI) techniques [8]. There has, however, been less work in this area than in some other areas of neuro-oncology; for example, there are fewer published papers on AI techniques for diagnosing post-treatment effects in IM than gliomas [9], despite IM being more common.

These techniques have typically focused on the imaging characteristics of the lesion in question, but the assessment should extend beyond simply the appearances of the lesion at a given timepoint. For example, RN preferentially involves the white matter rather than gray matter [10], and the risk is particular high in the corpus callosum [11]. Two additional features, which we have observed to be specific for RN, relate to anatomical boundaries and a change in the shape of the lesion. This case series describes these two features, and provides illustrative examples from the authors’ own clinical practice in which these features allowed a more confident diagnosis of RN.

## Case presentation

These cases were identified through the first author’s routine clinical practice at a tertiary level oncologic hospital. Institutional ethics committee approval was received for a waiver of patient consent. SRS was delivered either using a Varian TrueBeam linear accelerator with dynamic conformal arc therapy (DCAT) or with the Gamma Knife radiosurgery machine. The SRS schedules used in each case are specified in the respective figure legends.

### Anatomical boundaries

IM generally enlarge in one of two ways. Most commonly, IM enlarge in a relatively concentric manner, with progressive enlargement of a nodular lesion. Less commonly, metastases may extend along an anatomical surface, such as along the cortex or pial surface of the brain, or along the ependymal surface of the ventricular system. The frequency with which metastases enlarge in these two ways depends, in part, on the histology. For example, intracranial metastases from melanoma have a particular predilection to developing at the interface between the pia mater and the cortex [12], and curvilinear extension along the pial surface is a common occurrence. As such, the pattern of IM progression or recurrence is affected by the local anatomy. In contrast, the distribution of the radiation dose administered with SRS in general does not respect these anatomical boundaries, but rather is largely concentric around the treated lesion. This is illustrated by the isodose distributions as depicted in Figs. 3 and 6.

**Fig. 1:**
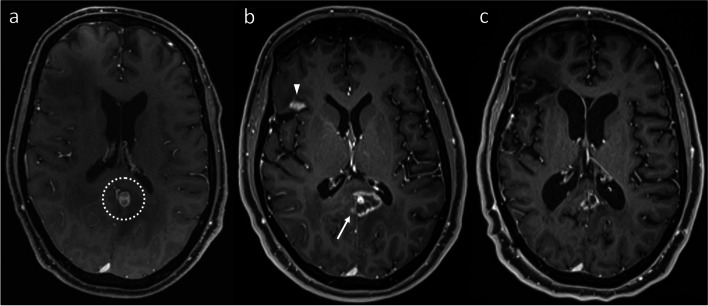
Axial post-contrast T1WI in a patient with an esophageal carcinoma metastasis (**a**, dotted circle) to the left cingulate gyrus, treated with 20 Gy single-fraction SRS. The lesion enlarged after SRS, with extension to the adjacent corpus callosum. On the 12-month post-SRS MRI (**b**), a small focus of enhancement developed in the right cingulate gyrus (arrow), discontinuous with the dominant lesion, which allowed a more confident diagnosis of RN. Note the “open ring” enhancement to the left of the falx cerebri; in contrast, if this was contiguous metastatic disease, abnormal enhancement of the falx cerebri would be expected. RN was supported by subsequent follow-up imaging, which demonstrated a change in the shape of the overall abnormality, with some regression of the dominant left-sided component, but further mild enlargement on the right. The appearances stabilized thereafter, and remain stable at most recent follow-up more than 12 months later. The patient also had a right frontal metastasis (**b**, arrowhead) treated with SRS, but this developed appearances suggestive of PD, prompting resection (which confirmed PD)

**Fig. 2:**
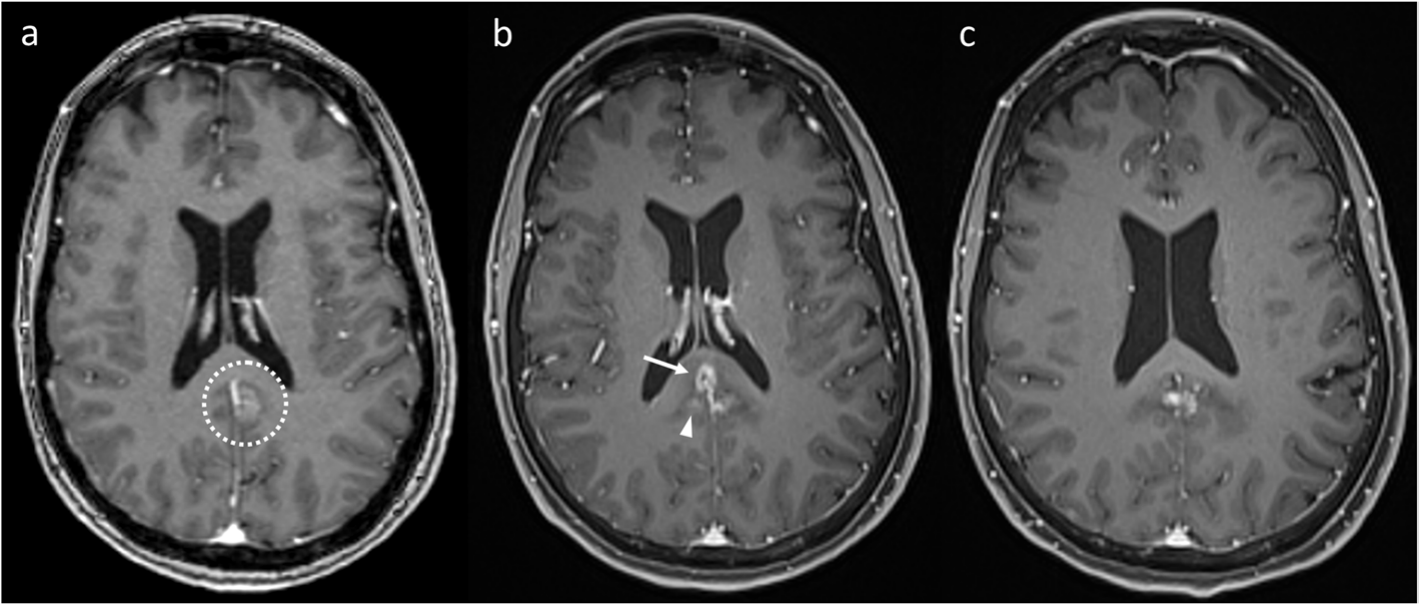
Axial post-contrast T1WI demonstrating a left cingulate gyrus metastasis (**a**, dotted circle) from a breast carcinoma primary, treated with 20 Gy single-fraction SRS. At 18-month follow-up (**b**), the metastasis had decreased in size, but a new peripherally-enhancing lesion had developed in the splenium of the corpus callosum (arrow); there was also new, subtle enhancement (arrowhead) in the right cingulate gyrus at this time. The abnormalities continued to evolve – another 19 months later (**c**), the splenial component had resolved, while the right cingulate gyrus component had mildly enlarged. The appearances stabilized thereafter, consistent with RN

**Fig. 3:**
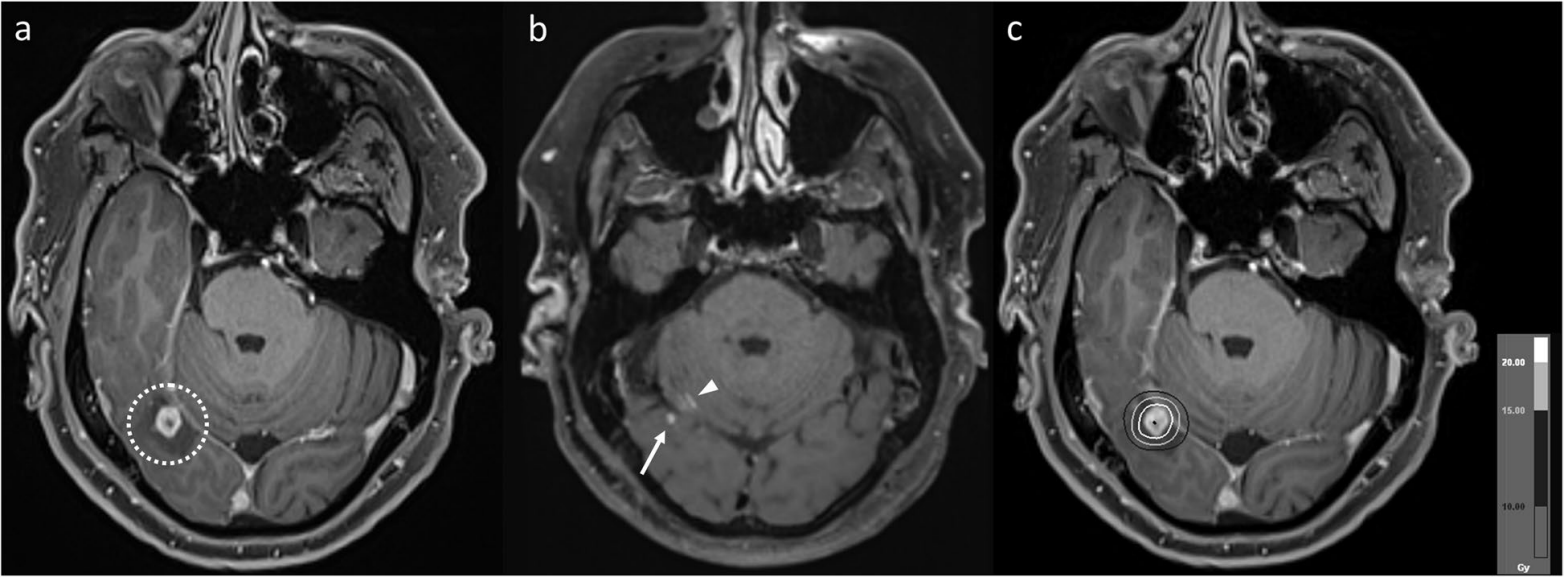
Axial post-contrast T1WI demonstrating a right occipital melanoma metastasis (**a**, dotted circle), which was treated with 20 Gy single-fraction SRS. The metastasis responded to SRS (arrow), but subtle linear enhancement developed in the adjacent right cerebellar hemisphere (**b**, arrowhead) four years later. Despite the linear appearance raising the possibility of leptomeningeal disease, this enhancement convincingly involved the cerebellar parenchyma rather than the folia. Geographic correlation of the area of enhancement with the high radiation dose region is illustrated by the 20 Gy, 15 Gy and 10 Gy isodose lines (in decreasing grayscale brightness) from the SRS plan (**c**). Ongoing stability of the cerebellar enhancement confirmed RN

**Fig. 4:**
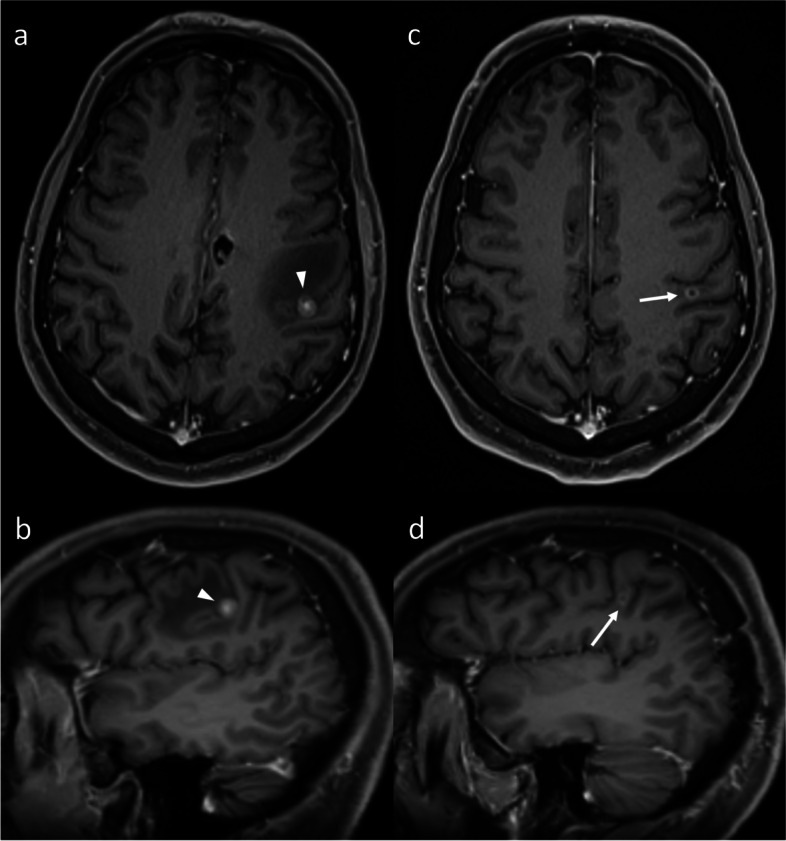
Axial (**a**) and sagittal (**b**) post-contrast T1WI shows a lung adenocarcinoma metastasis (arrowheads) to the left pre-central gyrus, which was treated with 20 Gy single fraction SRS. The sagittal plane best demonstrates that this arises anterior to the central sulcus, displacing it posteriorly. Ten months later (**c** & **d**), the lesion had resolved, but a peripherally-enhancing lesion (arrows) had developed in the adjacent post-central gyrus. This subsequently resolved without any specific treatment, confirming RN

**Fig. 5:**
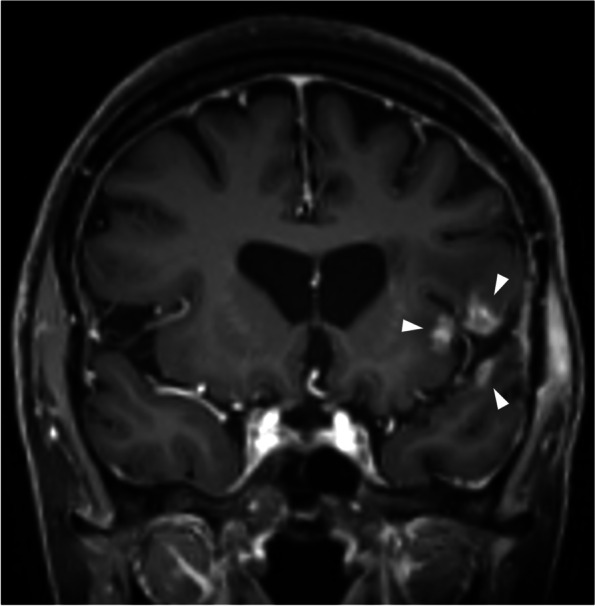
This patient had previously undergone resection of a left temporal melanoma metastasis and cavity SRS at an external institution (radiation dose-fractionation unclear), followed by intensity-modulated RT (20 Gy in 5 fractions) for localized leptomeningeal recurrence about two years later. Coronal post-contrast T1WI performed 15 months after the last episode of irradiation demonstrates separate (non-contiguous) areas of enhancement (arrowheads) around the left Sylvian fissure. The distribution, morphology and non-contiguous nature of this enhancement, conforming to the RT field, suggests RN, which was confirmed by subsequent regression

Several such anatomical boundaries exist in the brain. Firstly, this includes the major dural reflections: the falx cerebri (Figs. 1 and 2) and the tentorium cerebelli (Fig. 3). In our experience, the development of new enhancement on the other side of a dural reflection after SRS is strongly suggestive of RN rather than PD. A sulcus and CSF may provide a similar barrier (Figs. 4 and 5). In our experience of both above scenarios, the new enhancement occurring with RN preferentially involves the subcortical white matter, sparing the intervening cortex, and thus the new lesion can be anatomically distinct from, or non-contiguous with, the originally treated lesion. It is important, however, to ensure that there is no contiguous involvement of the intervening meninges, which would otherwise raise concern for PD.

**Fig. 6:**
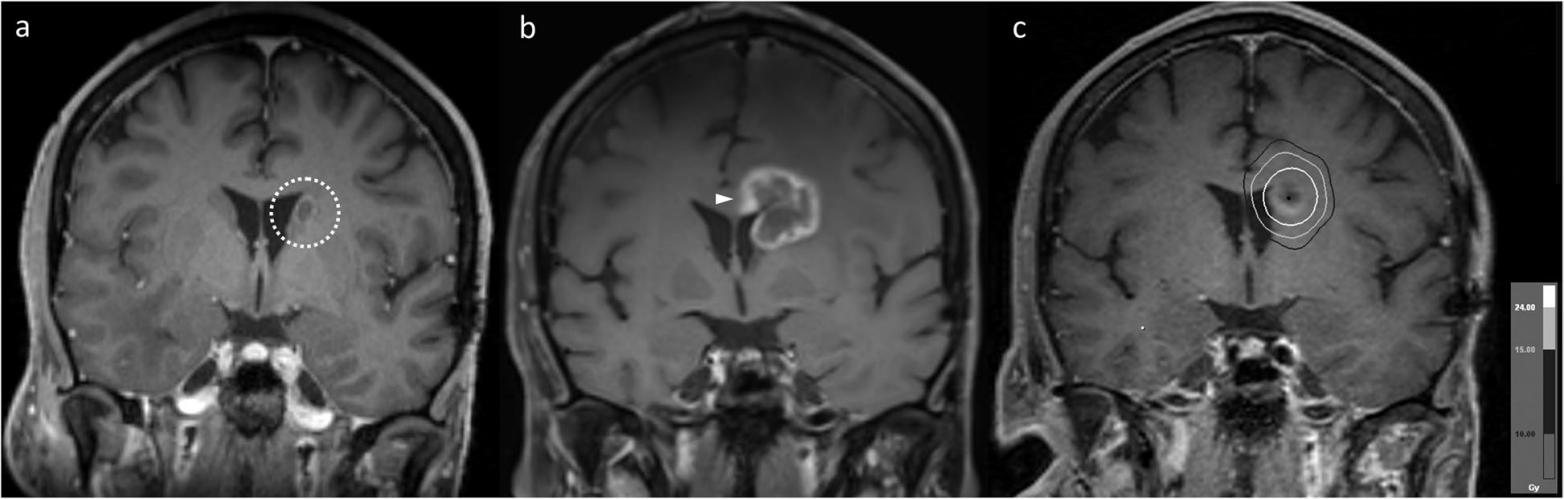
Coronal post-contrast T1WI in a patient with a left caudate nucleus metastasis (**a**, dotted circle) from a small cell lung cancer primary, treated with SRS (24 Gy in 3 fractions). The first post-treatment MRI at five months (**b**) shows that the lesion has substantially enlarged to involve the adjacent corpus callosum (arrowhead), wrapping around the adjacent lateral ventricle. Note the lack of enhancement along the ventricular surface, producing an “open ring” appearance. The eccentric morphology of the enlargement (with respect to the initial lesion), predominantly occurring superomedially across the ventricle, is more in keeping with RN than PD, which was confirmed by subsequent regression. There was also geographic correlation with the radiation high dose region, as illustrated by the 24 Gy, 15 Gy and 10 Gy isodose lines (in decreasing grayscale brightness) from the SRS plan (**c**)

**Fig. 7:**
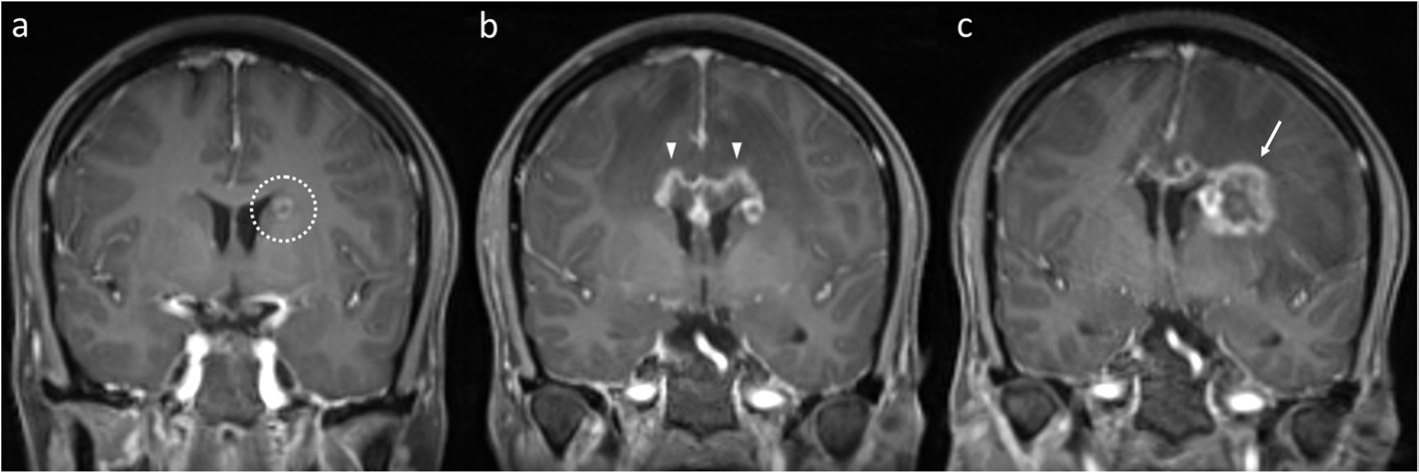
Coronal post-contrast T1WI demonstrates a metastasis (**a**, dotted circle) to the left caudate nucleus from breast cancer, treated with 27 Gy in 3 fractions of SRS at an external institution. Fifteen months after SRS (**b**), new extensive enhancement developed in the corpus callosum (arrowheads). Again note the absence of enhancement along the ventricular surface. At 2 years post-SRS (**c**), the callosal component has receded, but new enhancement (arrow) has development lateral to the treated lesion. This also subsequently receded without specific treatment, consistent with RN

Similar considerations exist for IM located close to the ventricular system (Figs. 6 and 7), though the MRI appearances differ to a degree, related to the anatomical differences compared to the more peripheral lesions discussed above. In contrast to more peripheral lesions, there is often no intervening grey matter between the treated IM and the white matter on the other side of the ventricle. As a result, in our experience, such RN lesions tend to develop in continuity, wrapping around the ventricle. Of note, and in contrast to metastatic disease, there is often no enhancement along the ventricle surface, producing an “open ring” appearance. This open ring appearance can also occur with treated metastases located away from the ventricular surface (see Fig. 1), related to the lower differential radiosensitivity of the cortex and the effect this in turn has on the MRI appearances (the ring potentially being open on the side of the cortex). Notably, in all our illustrated cases, the area of enhancement correlates geographically with the high radiation dose regions, as highlighted in Figs. 3 and 6.

### Change in shape

**Fig. 8:**
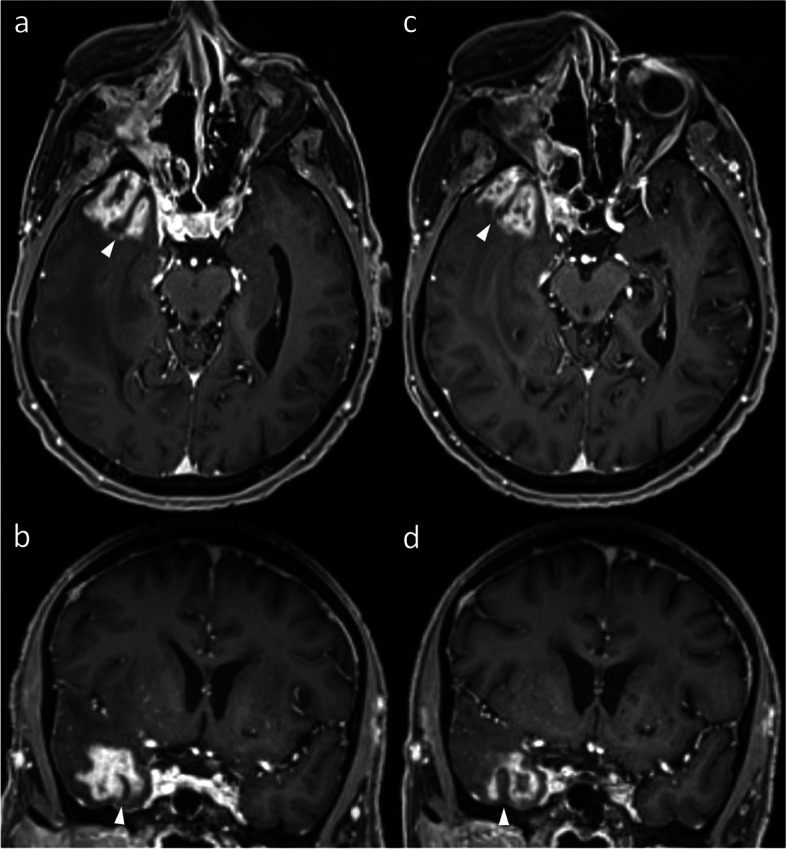
Axial (**a**) and coronal (**b**) post-contrast T1WI in a patient who was treated with a right orbital exenteration and adjuvant intensity-modulated RT (60 Gy in 30 fractions) for metastatic cutaneous squamous cell carcinoma to the medial canthus. Three years later, new enhancement developed in the right temporal lobe. While FET-PET (not shown) suggested true tumor progression, the suspicion of RN remained based on MRI, prompting a short-interval follow-up MRI (**c** & **d**). While the lesion is similar in size overall, multiplanar assessment reveals a change in the shape. The temporo-occipital sulcus (arrowheads) and adjacent cortex, which are spared, divide the overall lesion into two contiguous components: medial and lateral. While the medial component has enlarged, the lateral component has receded. The lesion continued to regress thereafter, confirming RN

The progression and evolution of IM tends to occur in a relatively simple manner. Prior to treatment, or if not responding to treatment, the entirety of an IM will enlarge, without regression of any particular components. While subsequent recurrence (and hence enlargement) may occur within only a portion of the initial lesion, often at its margin, any post-treatment regression will have already occurred. Thus, it would be very uncommon for different components of the same IM to enlarge and regress simultaneously. In contrast, RN is a more variable and dynamic process, affected by a variety of factors inherent to that particular part of the brain (or voxel), such as the dose received and the sensitivity to radiation effects. These factors vary from voxel to voxel, often leaving to a complex evolution of RN lesions. Radiologically, this can manifest as a change in the shape of the lesion, with different portions of the given lesions simultaneously enlarging and regressing (Figs. 7, and 8). Given that such evolution would not be expected for recurrent IM, such an appearance can be more confidently attributed to RN.

## Discussion

Given the challenges in accurately distinguishing between RN and PD, radiologists need to use all the tools at their disposal. A more nuanced approach beyond simply assessing the appearances of the lesion can provide greater diagnostic confidence. Anatomical boundaries, such as dural reflections and CSF, provide a barrier to enlargement of a metastasis, but not to RN. Thus, new or progressive enhancement on the other side of such a boundary suggests a diagnosis of RN. Another important tool at the radiologist’s disposal is follow-up imaging. The change in the shape of a lesion is a potential discriminating feature, and may manifest before the overall lesion has started to regress, or even despite overall enlargement of the lesion. Given the moderate incidence of RN after SRS in contrast to the low rate of local recurrence, a new or enlarging lesion at the site of previous SRS is statistically more likely to represent RN rather than PD. As a result, in our clinical practice we are hesitant to give a confident diagnosis of PD upon the initial identification of a new or enlarging lesion at the site of previous SRS. Instead, we have a low threshold for recommending short-interval follow-up imaging (supplemented by advanced sequences), at which time the features described may become evident.

While recent research into improving the diagnosis of RN has focused on augmenting the assessment with AI techniques, the subtle features described here are likely to be less amenable to accurate identification and characterisation by AI techniques. Going forward, it is important that the information assessed by AI-based techniques is not confined to the static imaging appearances alone, but also incorporates data on lesion location and temporal evolution, such as the features described here. The value of this approach has been shown in the use of deep learning for the prediction of glioma molecular subtype, in which cropping to the lesion led to lower prediction capability, attributed to the loss of information on tumor location and the correlation this has with molecular subtype [13, 14]. This highlights the need for an understanding of the expected imaging appearances, including the relevant sequences, when building and validating a robust AI model.

Nevertheless, emerging AI-based tools will remain only one part of the overall armamentarium, and the input of the neuroradiologist remains critical. Indeed, a multidisciplinary approach is important in these complex cases, and a variety of additional factors warrant consideration. These include the RT dose (for example if the patient has received repeat SRS or sequential whole-brain RT and SRS, both of which will increase the risk of RN) and neurosurgical considerations (for example the additional morbidity if the lesion were to enlarge). In addition, there have been some suggestions that simultaneous immunotherapy increases the likelihood of RN [15], though a recent systematic review suggests that there is no increased risk [16]. The time since SRS and the SRS technique used are also relevant, given suggestions that RN may occur earlier after Gamma Knife SRS than linear accelerator-based SRS [17].

We have encountered other similar cases in our clinical practice, but have been limited in the number which could be adequately presented, and thus specifically selected those which provide the most robust and broad demonstration of the concepts. Nevertheless, the selective nature of this case series is an inherent limitation. As such, we are unable to provide typical measures of a diagnostic test such as sensitivity and specificity. In our experience, these features are quite specific for RN, and this is supported by biological plausibility. However, our experiences also suggest that the sensitivity of these features is likely to be modest, which will provide some challenges to more formal evaluation. This is most intuitive for the anatomical boundaries feature, as only a minority of metastases are located in such a location. Nevertheless, it would be worthwhile including these features in future work assessing a multi-parametric approach to the diagnosis of RN, in particular determining the frequency of these features and confirming their suspected high predictive value.

## Conclusions

The distinction between PD and RN after SRS for IM is an increasingly common, but challenging, clinical scenario. While a variety of imaging techniques have been investigated, the value of any individual technique is limited. Assessment should, however, extend beyond simply the appearances of the lesion at a given timepoint, but also consider the tumor location and evolution. In particular, lesion development or enlargement across anatomical boundaries and a change in shape over time are both features which can increase confidence for a diagnosis of RN.

## Data Availability

Not applicable.

## Abbreviations

IM: Intracranial metastases
RT: Radiotherapy
SRS: Stereotactic radiosurgery
RN: Radiation necrosis
PD: Progressive disease / true tumor progression
MRI: Magnetic resonance imaging
PET: Positron emission tomography
FET: Fluorine-18-fluoroethyl-L-tyrosine
AI: Artificial intelligence
